# Effects of a psychological nursing intervention on prevention of anxiety and depression in the postpartum period: a randomized controlled trial

**DOI:** 10.1186/s12991-020-00320-4

**Published:** 2021-01-04

**Authors:** Hongling Liu, Yang Yang

**Affiliations:** Obstetrics Ward 3, Cangzhou Central Hospital, Xinhua West Road, Cangzhou, 061000 Hebei China

**Keywords:** Anxiety, Postpartum depression, Cognitive behavioral intervention

## Abstract

**Background:**

Anxiety and postpartum depression are the most common psychological problems in women after delivery. Cognitive behavior intervention has been reported to have an effect in the therapy of postpartum depression. This research aimed to investigate whether cognitive behavior intervention could prevent the pathogenesis of postpartum depression in primiparous women.

**Methods:**

In this randomized controlled trial, primiparous women who were prone to postpartum depression were recruited. Participates in the control group received routine postpartum care and those in the intervention group received both routine postpartum care and cognitive behavior intervention. Hamilton Depression Scale (HAMD), Hamilton Anxiety Scale (HAMA), Edinburgh Postpartum Depression Scale (EPDS) and Pittsburgh Sleep Quality Index (PSQI) were evaluated before and after the intervention.

**Results:**

In the intervention group, the post-intervention scores of HAMA, HAMD, EPDS and PSQI were all significantly lower than the baseline scores (*p* = 0.034, *p* = 0.038, *p* = 0.004, *p* = 0.014, respectively). The proportion of participants with postpartum depression in the intervention group (11.5%) was significantly lower than the control group (24.3%) after the 6-week intervention. Participants in the intervention group were significantly more satisfied with the care than those in the control group (*p* = 0.032).

**Conclusion:**

This research provided evidence that cognitive behavioral intervention in postpartum period could alleviate anxiety and depression in primiparous women, and inhibit the pathogenesis of postpartum depression.

*Trial registry* This clinical trial was registered in the Chinese Clinical Trial Registry (ChiCTR2000040076).

## Background

Pregnancy and delivery are two important physiological phenomena for women. In this process, the pregnant woman’s mood will change and become sensitive to psychological stimuli, leading to potential psychological problems [1], such as anxiety, depression and post-traumatic stress disorder [2]. It is demonstrated that the prevalence of post-traumatic stress disorder in women after delivery is 37.7% [3]. About 5–20% of mothers are influenced by post-delivery anxiety [4]. Postpartum depression accounts for 12.5% of psychologically related hospitalizations among women [5].

Impaired sleep duration and quality have been associated with concurrent mood disturbance and with increased risk of future mood problems during pregnancy and the postpartum period [6]. Anxiety is the mental reaction to either imagined or real threat. The symptoms of anxiety include smoking, high caffeine consumption, physical disease, poor nutrition and lack of sleep [7]. Accumulation of anxiety to a certain degree can cause disability [8]. In mothers, postpartum anxiety and depression inhibit oxytocin secretion and breast milk production [9]. As the most crucial postpartum stress complication, postpartum depression triggers increased vulnerability in both mothers and infants [10]. The role of the mother is affected by postpartum depression, and sometimes the mother’s attention in the child and the family is also compromised. Thus, postpartum depression is a major threat to the relationship between mother and infant [11]. Normally, depression will heal gradually after delivery if it is left untreated. Longer period of depression triggers complications that further enhances its severity [12].

Recently, evidence has indicated that interventions before and after delivery play a crucial role in reducing the risk of psychological problems in pregnant women. A meta‐analysis has demonstrated that several different interventions can be employed to alleviate the severity of postpartum anxiety and depression, including physical exercise, psychoeducation training, social support groups and cognitive behavior intervention [13]. A number of studies have shown that cognitive behavioral intervention and mental health care adjuvant therapy can effectively improve the condition of patients with postpartum depression. Clinical study has shown that cognitive behavioral intervention significantly reduces the scores of maternal postpartum depression scale and alleviates depression [14]. Another study has found that strengthening mental health care treatment for patients with postpartum depression can effectively reduce their depression self-rating scale scores and anxiety self-rating scale scores, and improve their quality of life after delivery [15].

In this research, we aimed to investigate whether psychological nursing intervention had a beneficial effect in preventing anxiety and depression in the postpartum period.

## Methods

### Study design and participants

This study was a randomized controlled trial conducted on primiparous women who had postpartum depression tendency during delivery. Participants in this study were recruited from Cangzhou Central Hospital by hospital administration staff who were not involved in any other part of the study. This research was approved by the ethics commitment of Cangzhou Central Hospital with informed consents signed by all the participants. This clinical trial was registered in the Chinese Clinical Trial Registry (ChiCTR2000040076).

843 primiparous women were recruited in this research. Inclusion criteria were: (1) primiparous women with full-term delivery, (2) having single healthy newborn baby, (3) having no obstetric diseases (eclampsia, placenta previa, and premature rupture of membranes, etc.), (4) having normal communication ability, and (5) having propensity for postpartum depression [Edinburgh Postpartum Depression Scale (EPDS) score ≥ 9 points but < 13 points]. Exclusion criteria included: (1) EPDS score < 9 points (having no postpartum depression tendency) or ≥ 13 points (having postpartum depression), (2) having serious underlying disease, including autoimmune disease, hypertension, or gestational diabetes, (3) having previous mental disease, and (4) having severe complications in mothers or infants. After exclusion, 260 patients remained in this research. Patients were centrally allocated (1:1) using concealed random allocation from a random number table generated by hospital IT staff who were not involved any other part of the study. A hospital nurse, who were not involved in any other part of the study, then assigned participants in different groups. All investigators, research staff, and the doctors treating the patients were masked to treatment allocation.

### Intervention

In the control group, participants received routine postpartum care, which involved the registration in community, postpartum life and dietary guidance provided by the hospital and community, guidance for women in maternal and newborn care, perineal care, breastfeeding, changing diapers, newborn bathing, and umbilical care, and answering questions from patients and families.

In the intervention group, in addition to routine postpartum care, participants received a 6-week cognitive behavior intervention, once a week, 1 h each time. Cognitive behavior intervention was composed of five different parts. First, a psychological evaluation was conducted on the parturient. Cognitive distortions from three perspectives were gently guided and corrected: the parturient to herself, to world she is in, and to the future. The parturient was made to understand some of the mental problems that might occur after childbirth and to face them correctly. Second, we assisted the mother to establish a self-activity plan (including control and joyful exercises, cognitive rehearsals, self-independent training, role-playing, and transfer techniques, etc.), and encouraged the parturient to regularly feedback to the doctor and make timely adjustment. Third, the mental health care, during which nurses communicated with mothers to establish a good nurse–patient relationship. Meanwhile, mentality, family and social background of mothers were analyzed to conduct targeted mental health education, stabilize postpartum anxiety, and provide appropriate emotional support. Soothing music was played to adjust mothers’ emotions and help them out of anxiety. Fourth, we enhanced the care for patients after delivery, guided their breastfeeding, assisted them to complete post-natal status change. Fifth, we actively communicated with the maternity husband and other family members, increased family members' social support for the mother, improved their negative emotional state and encouraged family members, especially the husband, to accompany and communicate with the patient and take care of the baby together.

### Measurements

Postpartum depression was identified through EPDS, which is a self‐report multiple-choice questionnaire with 10 items. The score of each item was from 0 to 3, and the total score was from 0 to 30. EPDS score ≥ 13 points was considered to indicate postpartum depression.

Anxiety symptoms were assessed by the Hamilton Anxiety Scale (HAMA). HAMA evaluation criteria were: < 7 points indicates anxiety-free; 7 ~  < 20 points indicates possible anxiety; 20 ~  < 29 points indicates anxiety; ≥ 29 points indicates severe anxiety.

Depression symptoms were assessed by the Hamilton Depression Scale (HAMD). HAMD evaluation criteria were: < 8 points indicates depression-free; 8 ~  < 20 points indicates possible depression; 20 ~  < 35 points indicates depression; ≥ 35 points indicates severe depression.

Sleep quality was assessed using the Pittsburgh Index Scale (PSQI). PSQI is a self-report questionnaire consisted of 19 items from seven subscales: sleep quality, sleep duration, sleep latency, sleep disturbance, sleep efficiency, sleep medication, and daily dysfunction. Each subscale had a score ranging from 0 to 3, and the total score was from 0 and 21. A higher score indicates worse sleep quality, and a total score > 7 indicates the presence of sleep disorders.

Nursing satisfaction was evaluated using the nursing satisfaction questionnaire developed by our hospital. The total score was 100 points, where ≥ 90 points indicates very satisfied; 75 ~  < 90 points indicates satisfied; 60 ~  < 75 points indicates basically satisfied; < 60 indicates dissatisfied.

### Statistical analysis

SPSS Statistics Version 22.0 software was employed for statistical analysis. Values were expressed as n (percentage, %) or mean ± SD. *p* values derived from unpaired t test or Mann–Whitney test as appropriate between intervention group and control group. *p* values derived from paired t test or Wilcoxon signed rank test as appropriate between baseline versus post-intervention. Chi-square test or Fisher’s exact test was used for assessing distribution of observations or phenomena between different groups. Statistical analysis was significant when *p* value < 0.05. Sample size was determined using established statistical power analysis. Differences between means of each compared treatment groups were divided by the standard deviation to determine the standardized effect size, then using 5% as significance level in Student t test and 90% power, the minimum required sample size was calculated, which was sufficient for our current sample size after consideration of dropout.

## Results

**Fig. 1:**
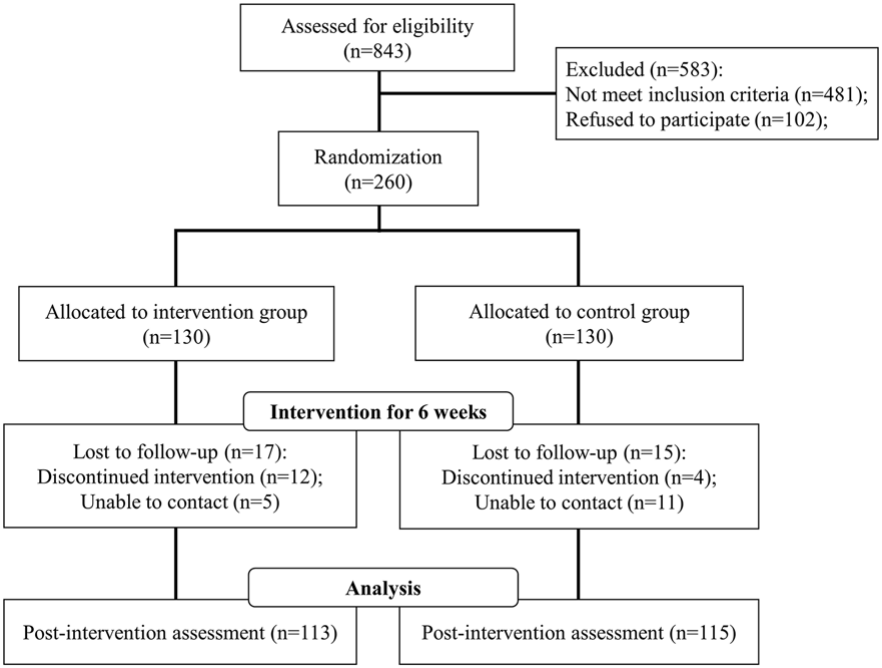
Research framework of this study

Research framework of this study is shown in Fig. 1. 843 primiparous women were assessed for eligibility. 102 patients refused to participate in this research and 481 patients did not meet the inclusion criteria. 260 participants were randomly assigned into the intervention group (*n* = 130) and the control group (*n* = 130). In the intervention group, 17 participants lost to follow-up, 12 of whom discontinued intervention and 5 were unable to contact. In the control group, 15 participants lost to follow-up, 4 of whom discontinued intervention and 11 were unable to contact. 113 participants in intervention group and 115 in the control group completed this research and their data were recorded and analyzed.

Table 1 shows the socio-demographic characteristics of the participants. No statistically significant differences were observed in socio-demographic characteristics between these two groups. The mean ages in the intervention group and the control group were 26.89 ± 4.12 and 27.31 ± 4.56 years, respectively. 60.2% of the women in the intervention group and 53.1% in the control group underwent vaginal delivery. In the intervention group, 16.8% of the patients graduated from college and 71.7% had work, while in the control group, the relative proportions were 21.7% and 64.3%, respectively. 76.1% of the women in the intervention group and 70.4% in the control group had no support of child caring. These data indicated that the participants in these two groups were homogenous.

**Table 1:** Socio-demographic characteristics of participants analyzed

Variable	Study group		*p*
	Intervention group (*n *= 113)	Control group (*n* = 115)	
Age (years)	26.89 ± 4.12	27.31 ± 4.56	0.273
BMI (kg/m^2^)			
18.5–24.9	48 (42.4%)	54 (46.9%)	0.789
25–29.9	42 (37.2%)	40 (34.8%)	
≥ 30	23 (20.4%)	21 (18.3%)	
Delivery			
Vaginal delivery	68 (60.2%)	61 (53.1%)	0.277
Caesarian section	45 (39.8%)	54 (46.9%)	
Education status			
Junior high school and below	23 (20.4%)	20 (17.4%)	0.601
Senior high school or polytechnic school	71 (62.8%)	70 (60.9%)	
College and above	19 (16.8%)	25 (21.7%)	
Husband education status			
Junior high school and below	15 (13.3%)	24 (20.9%)	0.310
Senior high school or polytechnic school	72 (63.7%)	66 (57.4%)	
College and above	26 (23.0%)	25 (21.7%)	
Working status			
Does not work	32 (28.3%)	41 (35.7%)	0.258
Works	81 (71.7%)	74 (64.3%)	
The sex of baby			
Girl	67 (59.3%)	57 (49.6%)	0.146
Boy	46 (40.7%)	58 (50.4%)	
Supports for caring the baby			
Yes	27 (23.9%)	34 (29.6%)	0.371
No	86 (76.1%)	81 (70.4%)	

Values were expressed as n (percentage, %) or mean ± SD. p values for each group were derived from either unpaired t test or Mann–Whitney test as appropriate. Chi-square test or Fisher’s exact test was used for assessing distribution of observations or phenomena between different groups

*BMI* body mass index

The incidence of postpartum depression in both groups were evaluated before and after the 6-week intervention. In this research, the patient was thought to have postpartum depression when the score of EPDS ≥ 13 points. As shown in Table 2, the incidence of postpartum depression in the control group (24.3%) was significantly higher than that of the intervention group (11.5%). This result showed that our intervention could effectively reduce the incidence of postpartum depression in women.

**Table 2:** Comparison of frequency distribution of postpartum depression between the two groups in different time points

Time	Variable	Study group		*p*
		Intervention group (*n* = 113)	Control group (*n* = 115)	
Baseline	Depressed	0 (0%)	0 (0%)	1.000
Post-intervention	Depressed	13 (11.5%)	28 (24.3%)	*0.015*

Values were expressed as n (percentage, %). Chi-square test or Fisher’s exact test was used for assessing distribution of observations or phenomena between different groups

Edinburgh Postnatal Depression Scale (EPDS) was used to diagnose the postpartum depression when the score ≥ 13

Italic value indicates the presence of statistical significance (*p* < 0.05)

We further investigated the satisfaction of the care in these two groups. In the intervention group, 51.3% of the patients were very satisfied with the care, 25.7% were satisfied, 15.9% were basically satisfied, and 7.1% were not satisfied. Meanwhile, in the control group, the relative proportions were 35.6%, 31.3%, 15.7%, and 17.4%, respectively, which were significantly lower than the intervention group (Table 3). Thus, the performance of intervention dramatically enhanced the satisfaction of the care.

**Table 3:** Comparison of satisfaction of the care between the two groups

Time	Study group		*p*
	Intervention group (*n* = 113)	Control group (*n* = 115)	
Very satisfied	58 (51.3%)	41 (35.6%)	*0.032*
Satisfied	29 (25.7%)	36 (31.3%)	
Basically satisfied	18 (15.9%)	18 (15.7%)	
Not satisfied	8 (7.1%)	20 (17.4%)	

Values were expressed as n (percentage, %). Chi-square test was used for assessing distribution of observations or phenomena between different groups

Italic value indicates the presence of statistical significance (*p* < 0.05)

Table 4 demonstrates the HAMA, HAMD, EPDS and PSQI scores of the women in both groups. After 6-week intervention, the HAMA, HAMD, EPDS and PSQI scores of the women in the intervention group were all dramatically lower than the control group. In the intervention group, the post-intervention scores of HAMA, HAMD, EPDS and PSQI were all significantly lower than the baseline scores. However, the scores of HAMD and PSQI were significantly elevated during the 6-week routine postpartum care in the control group. In primiparous women, the performance of cognitive behavior intervention together with routine postpartum care significantly reduced the incidence of anxiety and depression.

**Table 4:** Assessment of HAMA, HAMD, EPDS and PSQI before and after the intervention

Iteams	Study group		*p*
	Intervention group (*n* = 113)	Control group (*n* = 115)	
HAMA			
Baseline	13.24 ± 2.89	14.12 ± 3.21	0.134
Post-intervention	10.21 ± 2.16	15.55 ± 2.76	*0.007*
* p* value	*0.034*	0.089	
HAMD			
Baseline	14.36 ± 3.04	13.89 ± 2.93	0.117
Post-intervention	11.51 ± 2.85	16.58 ± 3.34	*0.021*
*p* value	*0.038*	*0.037*	
EPDS			
Baseline	11.24 ± 3.05	10.95 ± 2.75	0.217
Post-intervention	8.11 ± 2.18	11.45 ± 2.73	*0.008*
* p* value	*0.004*	0.283	
PSQI			
Baseline	9.12 ± 1.36	9.88 ± 1.53	0.172
Post-intervention	7.38 ± 1.09	12.03 ± 1.62	*0.006*
* p* value	*0.014*	*0.037*	

Values were expressed as mean ± SD. *p* values derived from paired t test or Wilcoxon signed rank test as appropriate between baseline versus post-intervention. *p* values derived from unpaired *t* test or Mann–Whitney test as appropriate between intervention group and control group

Italic values indicate the presence of statistical significance (*p* < 0.05)

## Discussion

This randomized, controlled clinical trial was performed to analyze the effect of psychological nursing intervention on postpartum depressive and anxiety symptoms. When compared with routine postpartum care, cognitive behavioral intervention significantly alleviated postpartum depressive and anxiety during delivery. The performance of cognitive behavioral intervention (psychological nursing intervention) was able to effectively reduce the incidence of postpartum depression in primiparous women who had a postpartum depression tendency.

In recent decades, puerperium has become an important stage after delivery, and has gradually attracted wide attention from obstetricians and pregnant women. During pregnancy and childbirth, parturient women often experience both physical and psychological changes, resulting in a significantly increased probability of mental problems after childbirth [16]. Clinical studies have shown that sudden changes in social roles of women after childbirth, coupled with rapid changes in social relationships and functions, exacerbate negative emotions such as anxiety and depression, leading to recurring unhealthy mental states [17]. Among these unhealthy mental states after delivery, postpartum depression is a common, disabling and treatable reproductive complication [18]. Worldwide statistical data indicate that 8–13% of primiparous women suffer from postpartum depression [19]. In the Greater China Region, the incidence of postpartum depression increases to around 20%, which becomes a great burden on medical system and society [19]. Recent research has demonstrated that postpartum depression patients could be divided into three different types: gradually cured (50.4%), partially improved (41.8%), and chronic severe (7.8%) [20]. Although most patients' postpartum depression symptoms can be relieved after 1 year, there are still some patients who remain depressed for a long time, and may even become more severe, leading to increased incidence of adverse events [21]. Thus, the identification of risk factors and the therapy for postpartum depression have become the focus of clinical work. The pathogenic factors of postpartum depression are complicated. Previous clinical study has illustrated the correlations between the pathogenesis of postpartum depression and altered levels of prenatal hormones, worrying about delivery and inadequate preparation for childbirth [22]. Another study further confirmed the contribution of limited postpartum health education, insufficient psychological support, and inability to adapt to postpartum roles to the occurrence of postpartum depression in the early postpartum period [21].

Cognitive behavioral intervention is commonly performed together with mental health care adjuvant treatment for clinical therapy of patients with depression [23]. As a complicated treatment system, cognitive behavioral intervention is composed of rational-emotive therapy, flooding therapy, systematic desensitization, relaxation training, social skills training and supportive treatment [24]. In cognitive behavioral intervention, the therapist evaluates the motivation and progression of treatment through observing the clinical performance, subjective needs and introspection of the patient, and adopts individualized treatment schemes depressed patients [25]. Evidence has shown that cognitive behavioral intervention and mental health care adjuvant therapy can effectively improve the condition of postpartum depression patients. Based on the results of several clinical studies, the score of EPDS and the levels of serum adrenaline and norepinephrine are significantly reduced by cognitive behavioral intervention [26]. Another study showed that enhanced mental health care treatment for postpartum depression patients effectively reduced the depression self-assessment scale score and anxiety self-assessment scale score, and improved the quality of life of postpartum patients [14]. In this research, the scores of HAMA, HAMD and EPDS in the intervention group were all significantly reduced by the 6-week cognitive behavior intervention. However, the scores of HAMA, HAMD and EPDS were elevated during the 6-week routine postpartum care in the control group. These results demonstrated that cognitive behavior intervention could alleviate postpartum anxiety and depression in primiparous women, whereas the routine postpartum care failed to do so. Studies have shown that negative emotional states are often closely related to the body's endocrine and metabolic functions. Negative emotions, such as anxiety and depression, can cause disturbances in the levels of related hormones in the maternal body, which can significantly compromise maternal sleep quality [27]. In this research, the significantly decreased PSQI score in the intervention group suggested the function of cognitive behavior intervention in improving the sleep quality of primiparous women. It is reported that cesarean section was associated with increased risk of postpartum depression [18]. Another study in Japan indicated that lower education level was associated with higher prevalence of postpartum depression and related symptoms [28]. Meanwhile, employment is considered as a protective factor for postpartum depression symptomatology [29]. Of note, in the current study, these factors showed no significant differences between the two groups.

Previous studies focused on the effect of cognitive behavioral intervention on the therapy of postpartum depression. However, whether cognitive behavioral intervention has an effect on the prevention of postpartum depression pathogenesis is still unknown. In this research, EPDS was employed for evaluating the degree of postpartum depression in primiparous women. Patients with EPDS score ≥ 9 and < 13 points were considered prone to postpartum depression. If EPDS score is ≥ 13 points, the patient is confirmed to have postpartum depression. To investigate the function of cognitive behavioral intervention in the prevention of postpartum depression pathogenesis, the EPDS scores of primiparous women who had postpartum depression tendency were evaluated after the 6-week intervention. Before the intervention, all participants in both groups had postpartum depression tendency. 6 weeks later, the proportion of participants with postpartum depression in the intervention group was dramatically lower than in the control group. This result indicated that cognitive behavioral intervention played a beneficial role in inhibiting postpartum depression pathogenesis in primiparous women. The results of nursing satisfaction questionnaire also illustrated the benefit of cognitive behavioral intervention in improving patients’ satisfaction of the care.

There are several limitations in the current study. First, the HAMD, HAMA and EPDS were screening tools for anxiety and depression, rather than diagnostic parameters. Although symptoms of anxiety and depression were analyzed in this research, clinical assessment and diagnosis of depression and anxiety were not performed. Another limitation in our study was the short follow-up period, and a follow-up period longer than 8 weeks in future studies may further confirm the results. Third, participants in the intervention group were encouraged to create self-activity plan and perform these practices. However, the actual time spent in home practice was not tracked. The performance of self-activity plan should be examined in future research.

## Conclusion

In summary, this research provided evidence that the performance of cognitive behavioral intervention in the postpartum period alleviated anxiety and depression in primiparous women and inhibited the pathogenesis of postpartum depression.

## Data Availability

Data could be obtained upon request to the corresponding author.
